# Resveratrol and caloric restriction prevent hepatic steatosis by regulating SIRT1-autophagy pathway and alleviating endoplasmic reticulum stress in high-fat diet-fed rats

**DOI:** 10.1371/journal.pone.0183541

**Published:** 2017-08-17

**Authors:** Shibin Ding, Jinjin Jiang, Guofu Zhang, Yongjun Bu, Guanghui Zhang, Xiangmei Zhao

**Affiliations:** 1 Department of Nutrition and Food Hygiene, School of Public Health, Xinxiang Medical University, Xinxiang, Henan Province, PR China; 2 Henan Collaborative Innovation Center of Molecular Diagnosis and Laboratory Medicine, Xinxiang Medical University, Xinxiang, Henan Province, PR China; 3 School of Public Health, Capital Medical University, Beijing, PR China; Universidade do Estado do Rio de Janeiro, BRAZIL

## Abstract

**Background:**

Studies have demonstrated that resveratrol (a natural polyphenol) and caloric restriction activate Sirtuin-1 (SIRT1) and induce autophagy. Furthermore, autophagy is induced by the SIRT1-FoxO signaling pathway and was recently shown to be a critical protective mechanism against non-alcoholic fatty liver disease (NAFLD) development. We aimed to compare the effects of resveratrol and caloric restriction on hepatic lipid metabolism and elucidate the mechanism by which resveratrol supplementation and caloric restriction alleviate hepatosteatosis by examining the molecular interplay between SIRT1 and autophagy.

**Methods and results:**

Eight-week-old male Wistar rats (40) were divided into four groups: the STD group, which was fed a standard chow diet; the HFD group, which was fed a high-fat diet; HFD-RES group, which was fed a high-fat diet plus resveratrol (200 mg/kg.bw); and the HFD-CR group, which was fed a high-fat diet in portions containing 70% of the mean intake of the HFD group rats. The groups were maintained for 18 weeks. Metabolic parameters, Oil Red O and hematoxylin-eosin staining of the liver, and the mRNA and protein expression of SIRT1, autophagy markers and endoplasmic reticulum(ER) stress-associated genes in the liver were assessed after the 18-week treatment. We found that resveratrol (200 mg/kg bw) and caloric restriction (30%) partially prevented hepatic steatosis and hepatocyte ballooning, increased the expression of SIRT1 and autophagy markers while decreasing ER stress markers in the liver and alleviated lipid metabolism disorder. Moreover, caloric restriction provided superior protection against HFD-induced hepatic fatty accumulation compared with resveratrol and the effects were associated with decreased total energy intake and body weight.

**Conclusion:**

We conclude that the SIRT1-autophagy pathway and decreased ER stress are universally required for the protective effects of moderate caloric restriction (30%) and resveratrol (a pharmacological SIRT1 activator) supplementation against HFD-induced hepatic steatosis.

## Introduction

Non-alcoholic fatty liver disease (NAFLD, characterized by steatosis) is one of the most common manifestations of chronic liver disease [1] and its prevalence is a serious and increasing clinical problem worldwide. An increasing amount of evidence supports the hypothesis of NAFLD as both cause and consequence of metabolic syndrome (MetS), and MetS is often characterized by lipid metabolic disorder and hepatic steatosis [2]. It is reported that 10~25% of NAFLD subjects will develop non-alcoholic steatohepatitis (NASH) [3], which typically progresses to cirrhosis and hepatic failure.

Resveratrol (3,4´,5-trihydroxystilbene), a dietary polyphenol found in grapes and red wine, has been demonstrated to exert protective effects on the liver against lipid metabolic disorder induced by a high-fat diet [4, 5]. Moreover, studies have reported the anti-steatosis effect of resveratrol in various rodent models of liver steatosis [6–8]. However, the exact effect and mechanism of resveratrol against steatosis (a phenotype of NAFLD) are still unclear. Caloric restriction (that is, the reduced caloric intake without malnutrition) extends the lifespan of a wide range of animals by reducing the incidence of metabolic diseases and arteriosclerosis [9]. Recently, some studies suggested that caloric restriction reversed metabolic dysfunction in the liver and induced a specific lipidomic and metabolomic reprogramming event in the mouse liver [10, 11]. This evidence indicates that caloric restriction may be a potential therapeutic approach to NAFLD, but the molecular mechanism of any such connection remains unknown. Autophagy is an intracellular degradation process that plays a critical role in cell survival, mitochondrial turnover, protein degradation, energy metabolism and hepatic lipid metabolism [12, 13]. Impaired autophagy has been described in several insulin-sensitive tissues, such as the liver [14], adipose tissue [15], and skeletal muscle [16], in the context of obesity. It has previously been shown that resveratrol ameliorates hepatic steatosis by inducing autophagy [17], and caloric restriction improves the metabolic conditions of high-fat diet-induced obese mice in an autophagy-correlated manner [18]. Moreover, resveratrol affects obesity-related complications in rodents by mimicking caloric restriction [5, 19], and caloric restriction mimetics the function of autophagy inducers *in vitro* and *in vivo* in rodents [20]. Sirtuin-1 (SIRT1) is an NAD^**+**^-dependent deacetylase that can be activated by caloric restriction or by pharmacological activators, particularly resveratrol [21]. Further studies have demonstrated that resveratrol mimics the effects of caloric restriction by activating the sirtuin system [22]. Previous data have shown that caloric restriction and resveratrol promote longevity through the SIRT1-dependent induction of autophagy both *in vitro* in human cells and *in vivo* in Caenorhabditis elegans [23], but the role of SIRT1 In the regulation of autophagy in the liver is still unclear. Endoplasmic reticulum (ER) stress is evident in the liver of obese animals [24] and is considered to play a key role in the regulation of lipogenesis as well as hepatic steatosis [25]. However, it is still unknown whether resveratrol, by mimicking the effects of caloric restriction prevents hepatic steatosis by regulating the SIRT1-autophagy pathway and alleviates ER stress.

Given that little is currently known about how resveratrol and caloric restriction impact on autophagy and ER stress in the liver, the effects of resveratrol and caloric restriction on the autophagic mechanism of hepatic lipid metabolism and hepatic ER stress will be of interest. In this study, we aimed to investigate the effect of resveratrol supplementation and caloric restriction treatment on high-fat diet-induced NASH and further explored whether they contribute to hepatoprotection through the regulation of the SIRT1-autophagy pathway as well as decreased ER stress.

## Materials and methods

### Chemicals and reagents

Resveratrol (3,4´,5-trihydroxystilbene) was purchased from Sigma Chemical Company. Assay kits for serum total cholesterol (TC), total triglycerides (TG), high density lipoprotein cholesterol (HDL) and low density lipoprotein cholesterol (LDL) were obtained from BIOSINO Bio-technology and Science, Inc. (Beijing, China). TRIzol was obtained from Invitrogen Inc. (Carlsbad, CA, USA) and a real-time quantitative PCR kit was purchased from TAKARA Bio Inc. (Otsu, Shiga, Japan). LC3B antibody, p62 antibody, GRP78 antibody and CHOP antibody were obtained from Cell Signaling Technology (Billerica, MA, USA); SIRT1 antibody and secondary antibody were purchased from Abcam (Cambridge, UK). All other reagents were of analytical grade.

### Animals

Eight-week-old male Wistar rats (190–207 g) were purchased from the Vital River Laboratory Animal Technology Co., Ltd. (Beijing, China). The rats were housed in pairs of two at a controlled temperature (20–22°C) under a 12 h/12 h light/dark cycle. This study was carried out in strict accordance with the recommendations in the Guide for the Care and Use of Laboratory Animals by the National Institutes of Health. The protocol was approved by the Committee on the Ethics Animal Experiments of Xinxiang Medical University. All procedures were performed under sodium pentobarbital anesthesia, and all efforts were made to minimize suffering.

### Resveratrol supplementation and caloric restriction

After a one-week acclimation period, the rats were randomly distributed into four groups (n = 10 for each group) and treated for 18 weeks as follows: (i) the standard chow diet group (STD) was fed a standard rodent chow diet; (ii) the HFD group was fed a high-fat diet (HFD); (iii) the HFD-RES group was fed a high-fat diet and resveratrol (200 mg/kg.bw); (iv) the HFD-CR group was fed a high-fat diet in portions containing 70% of the mean intake of the HFD group rats. The food intake of the rats in HFD-CR group was adjusted each week according to the intake of the HFD group during the preceding week [10]. The macronutrient compositions of the standard chow diet and the high-fat diet are shown in Table 1, and the ingredient composition of the HFD fed to rats was as follows (w/w): standard chow, 60%; custard powder, 8%; lard, 12%; sugar, 12%; peanut podwer, 6%; and milk, 1%[26]. According to previous reports [27, 28] and the safe dose that is comparable with doses treated in clinical trials [29, 30], as well as after due dose translation [31], the dose of resveratrol (200 mg/kg bw) was selected in our study. The details of the resveratrol supplementation method are as follows: resveratrol was mixed with the high-fat diet (5 g/rat) as the test diet. In the morning, rats were provided with the test diet, the high-fat diet, or the standard chow diet and then sufficient standard chow diet or high-fat diet was provided after the test diet was completely consumed. The body weight of each rat was recorded weekly, and their food intake was weighed every day. After 18 weeks of treatment and an overnight fast, the animals were decapitated swiftly. The liver and fat tissues were then extracted, immediately frozen in liquid nitrogen, and stored at -80°C until further analysis.

**Table 1:** The macronutrient composition of the standard chow diet (STD) and the high-fat diet (HFD).

	Fat (%)	Carbohydrates (%)	Protein (%)	Total energy (kcal/g)
STD	13.68	64.44	21.88	3.29
HFD	41.26	39.61	19.13	4.59

### Energy intake assessment

The total food intake during the experiment was calculated according to the daily food intake records; the total energy intake of each of the four groups over the 18-week period was the total food intake multiplied by the provided energy of the appropriate diet.

### Determine of serum metabolic parameters and hepatic lipid parameters

Serum was obtained after centrifugation of whole blood (845 g for 10 min at 4°C). Total cholesterol (TC), total triglyceride (TG), high-density lipoprotein (HDL) and low-density lipoprotein (LDL) were measured using enzymatic kits (BIOSINO Biotechnology and Science, Inc.). Lipids were extracted from liver tissue (100 mg) using the procedure described by Loison *et al* [32], and then the TG and TC levels were measured using enzymatic colorimetric assays (BIOSINO Biotechnology and Science, Inc.).

### Histopathological analysis

Liver samples were fixed in 4% paraformaldehyde, dehydrated, and embedded in paraffin. Sections (5 μm thick) were prepared and stained with hematoxylin-eosin (HE) stain for histological analysis under a light microscope. To evaluate fat deposition, we embedded liver samples in Tissue-Tek® OCT compound (Sakura FineTek USA, Torrance, CA, USA) and then stained them with Oil Red O. Hepatic steatosis was assessed by point counting methods as mentioned previously [33, 34]. The detailed method is as follows: five slices of HE per animal were performed and 10 microscopic fields were selected at random for estimating volume density (Vv) of liver steatosis by point-counting methods.

### RNA isolation and real-time polymerase chain reaction

Total RNA was extracted from the liver using TRIzol reagent (Invitrogen, USA), according to the manufacturer’s instructions. Reverse transcription (RT) to produce cDNA was carried out using a PrimeScript™ RT Master Mix (Perfect Real Time) (TAKARA Bio Inc., Otsu, Shiga, Japan) according to the manufacturer’s instructions. Reaction mixtures (50 μL), each containing 50 μg of total RNA were heated for 20 min at 37°C. The mRNA expression of *LC3*, *Beclin1*, *p62*, *SIRT1*, *PERK*, *GRP78*, *CHOP* and *β-actin* in liver tissue was determined using the SYBR Green detection system on an ABI PRISM 7900 machine (Applied Biosystems) with the following conditions: one cycle, 95°C, 5 s; 40 cycles, 95°C, 10 s; and 57°C, 30 s. The primer sequences used for real-time PCR are listed in Table 2. The 2^-ΔΔCT^ method was adopted for the data analysis, and all sample values were normalized to expression of the endogenous reference gene β-actin.

**Table 2:** Primer sequences used for real-time PCR.

Gene	Forward primer (5`-3`)	Reverse primer (5`-3`)	Accession no.
*LC3*	ATAGAGCGATACAAGGGTG	AGGAAGAAGGCTTGGTTA	NM_022867
*Beclin 1*	GCTTCCCTGGTAGGTGTCA	CTTTCTTCTGCCGTTCCTT	NM_053739
*p62/SQSTM1*	CGGAAGTCAGCAAACC	ATGCGTCCAGTCGTCA	NM_175843
*SIRT1*	GACGACGAGGGCGAG GAG	ACAGGAGGTTGTCTCGGTAGC	XM_017601788
*PERK*	TTTAAGGGAGTGGCTTGATTT	AGGTTGGGATTGTTGGTGTC	NM_001191926
*GRP78*	CAGATCTTCTCCACGGCTTC	GCAGGAGGAATTCCAGTCAG	AY_216677
*CHOP*	GCATGAAGGAGAAGGAGCA	CTTCCGGAGAGACAGACAG	NM_024134
*β-actin*	CGTGCGTGACATTAAAGAG	TTGCCGATAGTGATGACCT	NM_031144

LC3, microtubule-associated protein 1A/1B-light chain 3; SIRT1, sirtuin 1; PERK, protein kinase RNA-like endoplasmic reticulum kinase; GRP78, glucose-regulated protein 78/immunoglobulin heavy-chain binding protein; CHOP, CCAAT/enhancer-binding protein-homologous protein.

### Protein extraction and Western blot analysis

The protein expression levels of LC3, p62, SIRT1, GRP78 and CHOP in the liver were measured by Western blot analysis. The liver tissue proteins were extracted using a commercial protein extraction kit (Beyotime, Nantong, China) according to the manufacturer’s instructions. The protein concentration was measured using a BIO-RAD DC Protein Assay Reagent (Bio-Rad, Hercules, CA, USA). Proteins separated by 12% or 10% sodium dodecyl sulfate-polyacrylamide gel electrophoresis were transferred to PVDF membranes. The blots were incubated with anti-LC3B antibody (diluted 1:1000), anti-p62 antibody (diluted 1:1000), anti-CHOP antibody (diluted 1:1000), anti-GRP78 antibody (diluted 1:1000) and anti-SIRT1 (diluted 1:1000) antibody overnight at 4°C, and then incubated with HRP-conjugated secondary antibodies. β-Actin (1:10000, Sigma-Aldrich Chemical Company, St. Louis, Missouri) was used as a loading control. Then, the proteins were visualized with an ECL detection system (Syngene, Cambridge, UK). Densitometry analysis was performed using ChemiDoc Quantity One software (Bio-Rad Laboratories).

### Statistical analyses

Statistical analyses were carried out using SPSS13.0 statistical software (SPSS, Chicago, IL). Data are expressed as the mean ± SD. Statistical differences between two groups were evaluated by Student’s t-test; comparisons among groups were analyzed for statistical significance by one-way analysis of variance (ANOVA), followed by post hoc analysis (Bonferroni’s test). All *P* values were from two-tailed tests, and *P* < 0.05 was considered significant.

## Results

### Body weight, liver weight/body weight ratio and total energy intake

**Fig 1:**
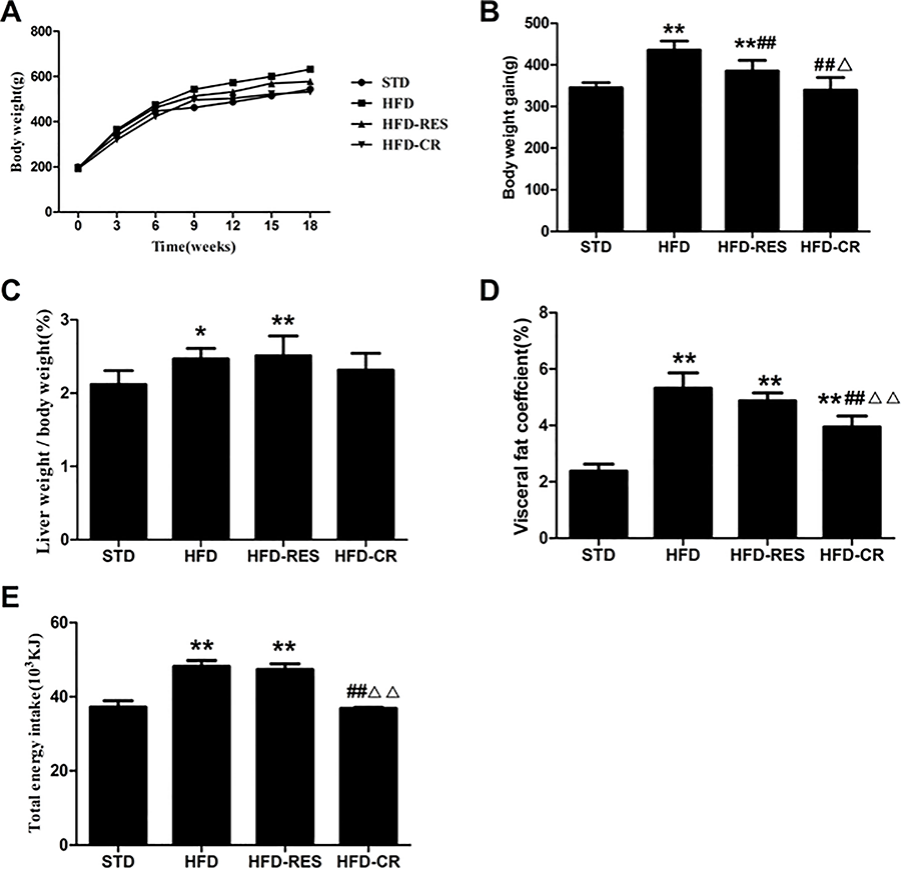
Effects of RES and caloric restriction on body weight curve, body weight gain, liver weight/body weight and total energy intake in rats. (A) Body weight curve (n = 10 per group). (B) Body weight gain (n = 10 per group). (C) Liver weight/body weight (n = 10 per group). (D) Visceral fat coefficient (n = 10 per group). (E) Total energy intake (n = 5 per group). ^*^, *P*<0.05 and **, *P*<0.01 compared with the STD group; ^#^, *P*<0.05 and ^##^, *P*<0.01 compared with the HFD group; ^△^, *P*<0.05 and ^△△^, *P*<0.01 compared with the HFD-RES group. Data are expressed as the mean ± SD.

As shown in Fig 1, significant differences in body weight and body weight gain were found between the STD group and the high-fat-fed groups (*P*<0.01 or *P*<0.05). Moreover, treatment with resveratrol (RES) and caloric restriction (CR) significantly decreased the body weight gain of high-fat fed mice (*P*<0.01); however, no difference in body weight gain was observed between the STD group and the HFD-CR group (*P*<0.05). The liver weight/body weight ratios of the HFD group and the HFD-RES group was significantly higher than that of the STD group (*P*<0.01 and *P*<0.05). Compared with the STD group, the visceral fat coefficient was markedly increased in the HFD group, HFD-RES group and HFD-CR group (*P*<0.01), and there was no significant difference in visceral fat coefficient between the HFD group and the HFD-RES group. In addition, caloric restriction reduced the visceral fat coefficient of HFD-fed rats with respect to the HFD group (*P*<0.01) and the HFD-RES group (*P*<0.01). Significant differences in total energy intake were observed between the STD group and the HFD group (*P*<0.01; and were also observed between the STD group and the HFD-RES group (*P*<0.01). However, no significant changes were observed in total energy intake between the STD group and the HFD-CR group (*P*>0.05). These data indicated that RES supplementation has no effect on total energy intake, while CR treatment can decrease body weight and liver weight/body weight ratio by regulating total energy intake.

### Lipid parameters in the serum and liver

**Fig 2:**
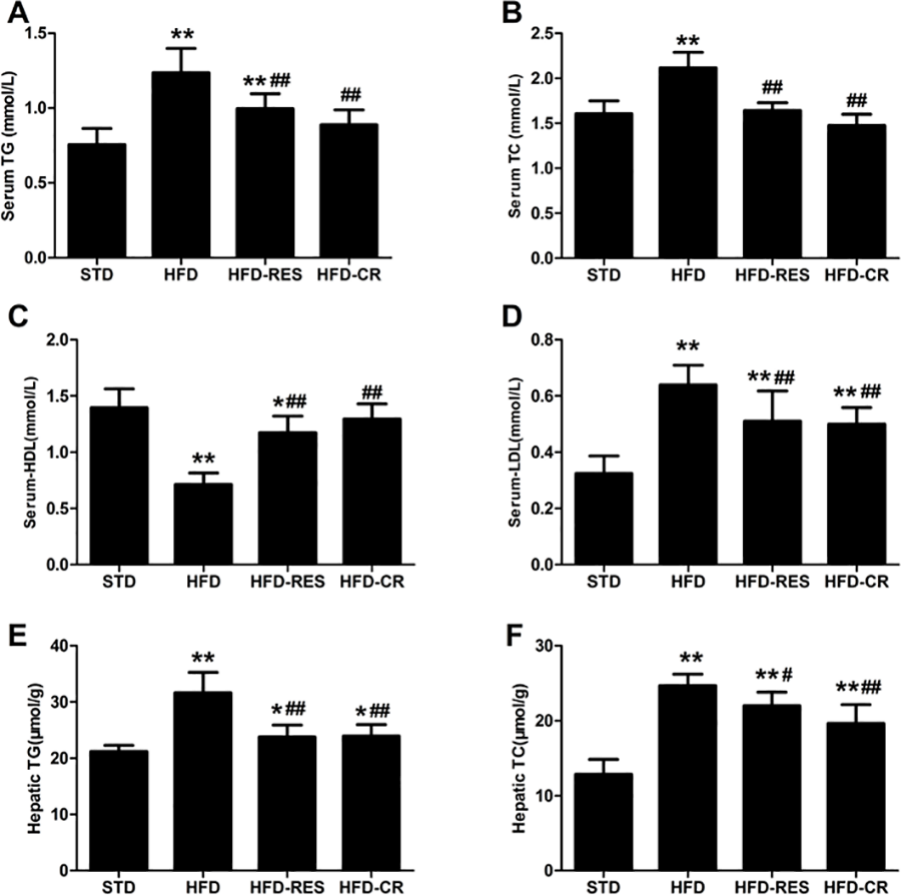
Lipid parameters in the serum and liver of rats (n = 8–10 per group). (A) Serum total triglycerides. (B) Serum total cholesterol. (C) Serum HDL. (D) Serum LDL. (E) Hepatic total triglycerides. (F) Hepatic total cholesterol. ^*^, *P*<0.05 and **, *P*<0.01 compared with the STD group; ^#^, *P*<0.05 and ^##^, *P*<0.01 compared with the HFD group. Data are expressed as the mean ± SD.

As shown in Fig 2, compared with the STD group, the TG and TC levels in the serum and liver were significantly increased in the HFD-fed groups (*P*<0.01). Furthermore, the serum LDL levels were markedly increased in the HFD-fed groups (*P*<0.01). After 18 weeks of treatment, the serum levels of TG and TC were significantly reduced in both the RES group and the CR group (*P*<0.01 or *P*<0.05) compared with the HFD group. The elevated serum LDL levels of the HFD-fed rats were significantly lowered by treatment with RES and CR (*P*<0.01). With regard to HDL, a significant difference was observed between the STD group and the HFD group (*P*<0.01) and compared with the HFD group, the levels of HDL were significantly increased in the RES group and the CR group (*P*<0.01). There was no effect of RES or CR treatment on TG, TC, HDL or LDL levels in the serum or on TG and TC levels in the liver of HFD-fed rats (*P*>0.05).

### Morphology and lipid accumulation in the rat liver

**Fig 3:**
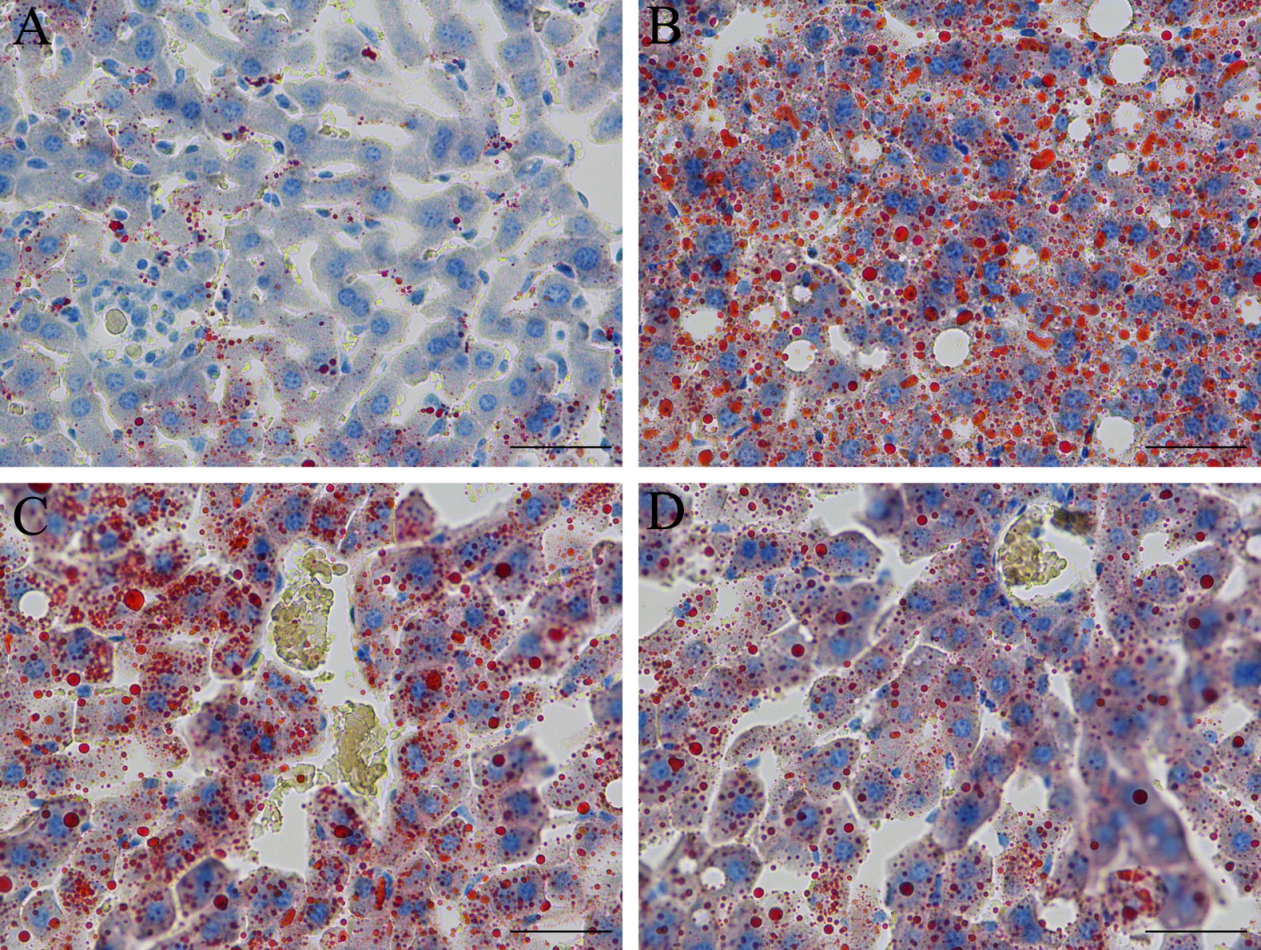
Oil Red O staining observation of rat liver tissue (original magnification: ×400, scale bars = 50 μm). (A) STD group. (B) HFD group. (C) HFD-RES group. (D) HFD-CR group.

**Fig 4:**
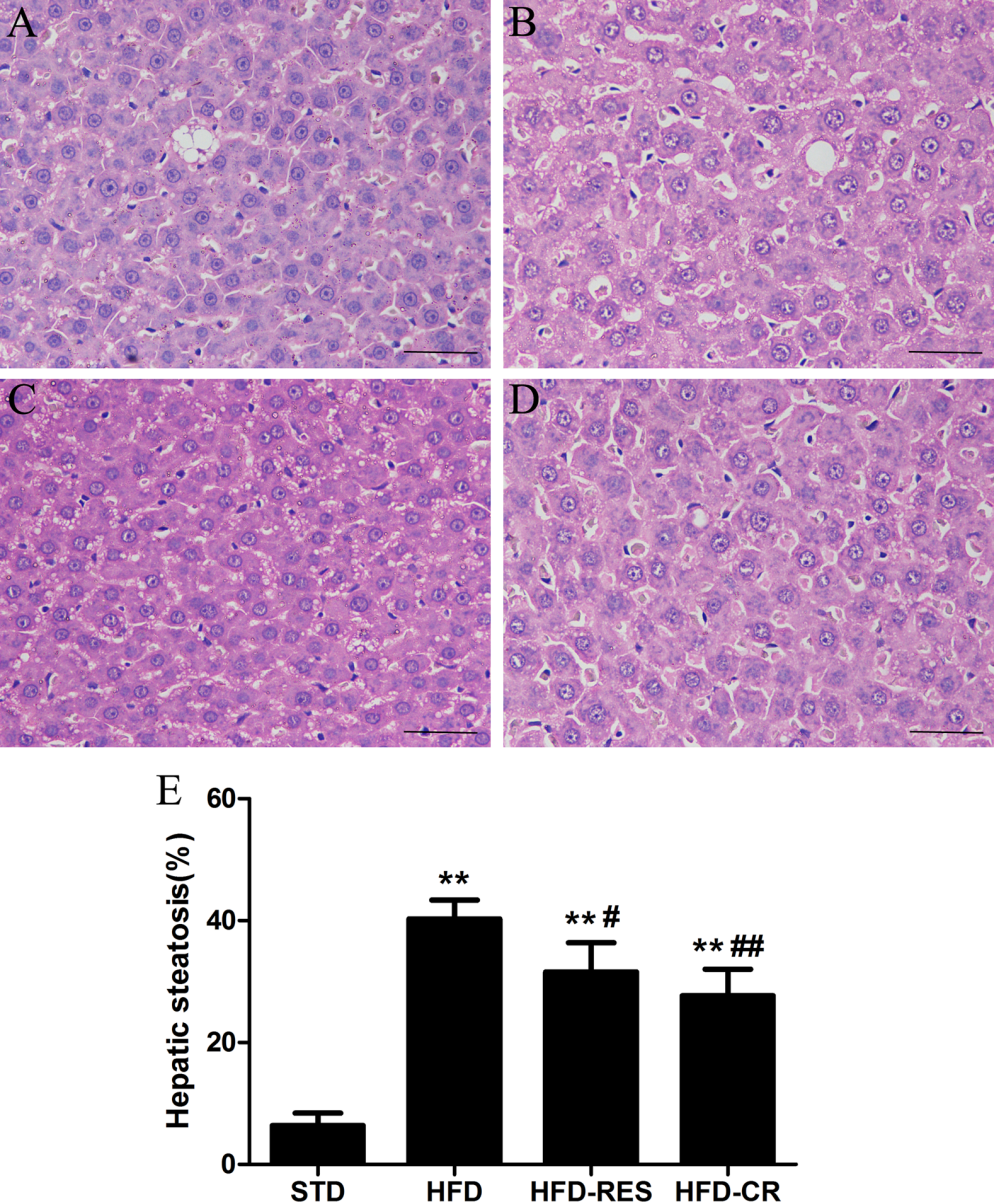
Hematoxylin and eosin staining observation of rat liver tissue (original magnification: ×400, scale bars = 50 μm). (A) STD group. (B) HFD group. (C) HFD-RES group. (D) HFD-CR group. (E) Volume density of quantitation of hepatic steatosis (n = 5 per group). **, *P*<0.01 compared with the STD group; ^#^, *P*<0.05 and ^##^, *P*<0.01 compared with the HFD group.

To evaluate the effects of RES treatment and CR on hepatic steatosis, we stained liver tissue with Oil Red O stain (×400) and hematoxylin-eosin (HE, ×400) stain to visualize lipid accumulation under all treatment conditions. As shown in Fig 3, compared with the STD group, the lipid accumulation in the liver was increased in the HFD group; moreover, treatment with RES and CR for 18 weeks decreased hepatic intracellular lipid accumulation in rats. As shown in Fig 4, extensive macrovesicular steatosis surrounding the perisinusoidal areas and microvesicular steatosis were observed in high-fat diet-fed rats. However, RES and CR treatment significantly decreased the accumulation of intracellular lipid droplets in HFD-fed rats.

### Hepatic mRNA expressions in rats

**Fig 5:**
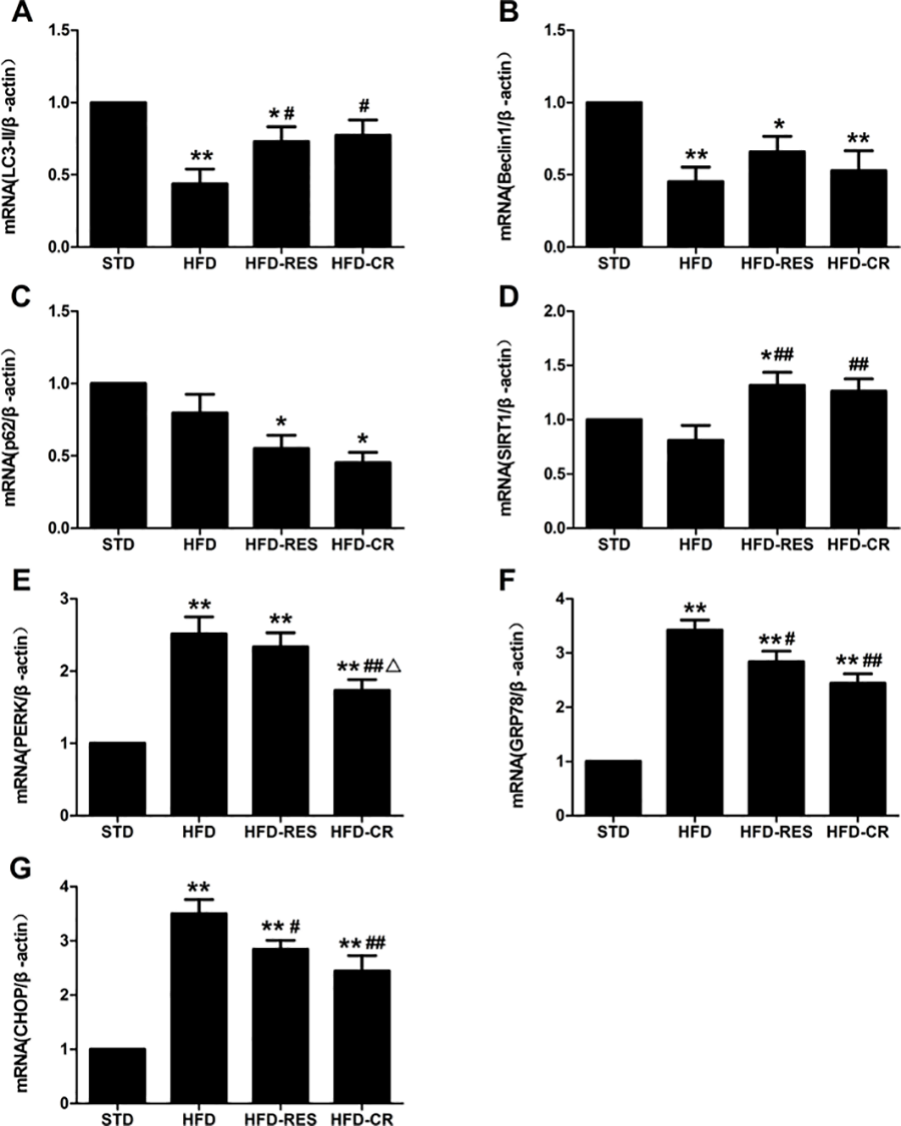
Changes of expression autophagy relative genes, ER stress genes and SIRT1 gene in the rat liver (n = 3 per group). (A) *LC3*. (B) *Beclin 1*. (C) *p62*. (D) *SIRT1*. (E) *PERK*. (F) *GRP78*. (G) *CHOP*. ^*^, *P*<0.05 and **, *P*<0.01 compared with the STD group; ^#^, *P*<0.05 and ^##^, *P*<0.01 compared with the HFD group; ^△^, *P*<0.05 compared with the HFD-RES group. Data are expressed as the mean ± SD.

The level of hepatic expression of *SIRT1*, autophagy marker genes and ER stress-associated genes by RES and CR treatment was assessed using real-time PCR (Fig 5). The expressions of *LC3* and *Beclin1* mRNA were decreased in the HFD group compared to the STD group (*P*<0.01) and the increased mRNA level of *LC3* was observed in the HFD-RES group and HFD-CR group compared to the HFD group (*P*<0.05). However, no significant difference of the mRNA expression level of the *LC3* was observed between in the HFD-RES and HFD-CR group (*P*>0.05). The mRNA expression of *p62* was slightly decreased in the HFD group compared to the STD group (*P*>0.05) and *p62* mRNA was decreased in the HFD-RES group and HFD-CR group compared to the STD group (*P*<0.05), but detected no change between the HFD-RES group and the HFD-CR group (*P*>0.05). In addition, no significant difference was observed between the STD group and HFD group in the level of *SIRT1* mRNA expression (*P*>0.05). Moreover, both RES and CR treatment significantly increased the mRNA expression of *SIRT1* in HFD-fed rats (*P*<0.01 and *P*<0.05). Compared to STD group, the mRNA levels of *PERK*, *GRP78* and *CHOP* in liver were significantly increased (*P*<0.01); both resveratrol supplementation and caloric restriction significant decreased the mRNA levels of *GRP78* and *CHOP* in liver compared to the HFD group (*P*<0.01 or *P*<0.05). There was a significant decrease of *PERK* level in the HFD-CR group compared to the HFD group and HFD-RES group (*P*<0.01 or *P*<0.05).

### The protein expressions of liver in rats

**Fig 6:**
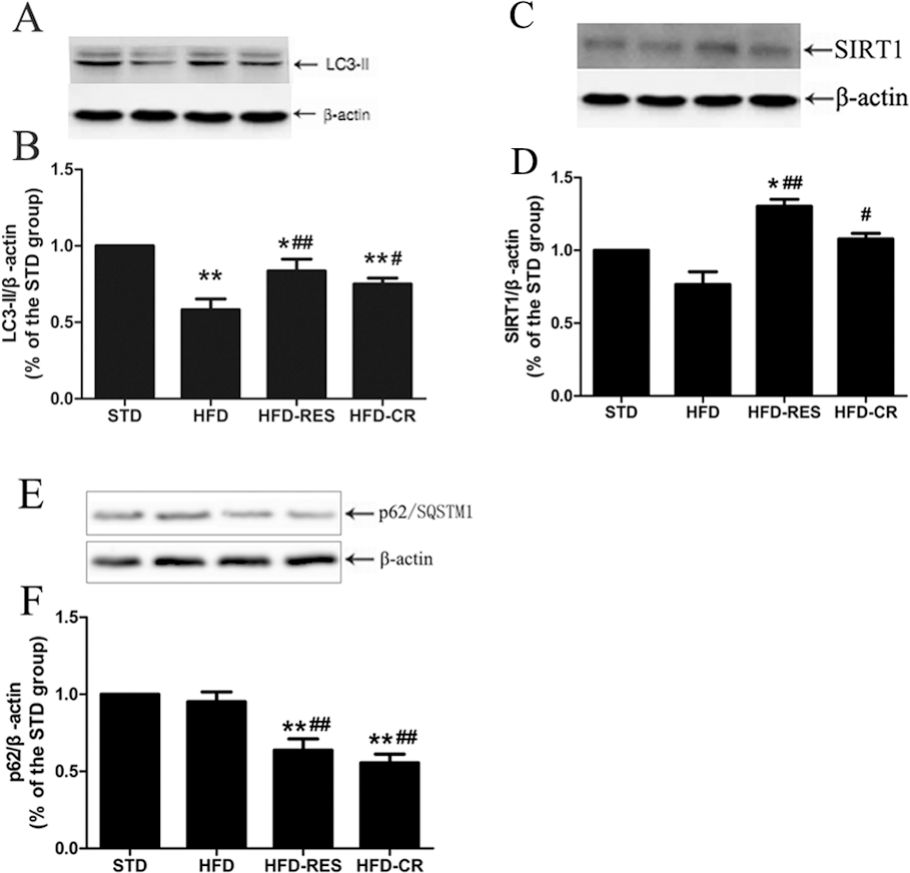
Effects of RES and CR on the protein levels of autophagy markers and SIRT1 in the liver (n = 3 per group). (A) Western blotting results for LC3 protein. (B) Quantitative analysis of LC3-II band densities. (C) Western blotting results for SIRT1 protein. (D) Quantitative analysis of SIRT1 band densities. (E) Western blotting results for p62 protein. (F) Quantitative analysis of p62 band densities. ^*^, *P*<0.05 and **, *P*<0.01 compared with the STD group; ^#^, *P*<0.05 and ^##^, *P*<0.01 compared with the HFD group. Data are expressed as the mean ± SD.

**Fig 7:**
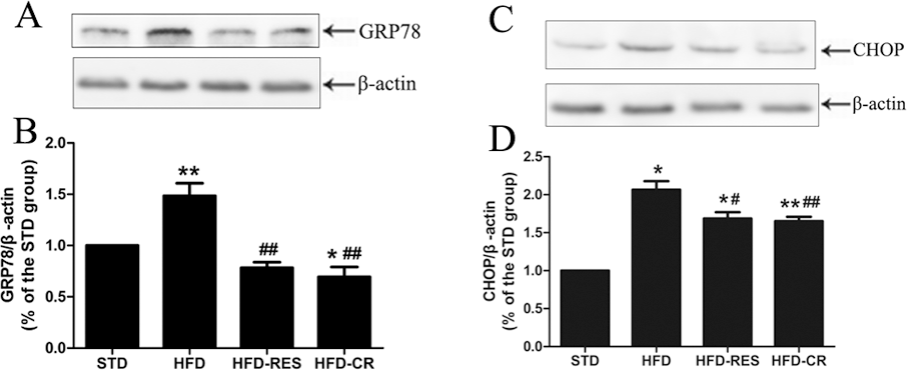
Effects of RES and CR on the protein levels of endoplasmic reticulum stress markers in the liver (n = 3 per group). (A) Western blotting results for CHOP protein. (B) Quantitative analysis of CHOP band densities. (C) Western blotting results for GRP78 protein. (D) Quantitative analysis of GRP78 band densities. ^*^, *P*<0.05 and **, *P*<0.01 compared with the STD group; ^#^, *P*<0.05 and ^##^, *P*<0.01 compared with the HFD group. Data are expressed as the mean ± SD.

The protein expressions of SIRT1, autophagy markers (LC3 and p62/SQSTM1), ER stress markers (CHOP and GRP78) in the liver were detected (Figs 6 and 7). Rats fed with HFD showed decreased LC3-II protein expression in the liver compared with STD group (*P*<0.01), and the treatment of RES and CR markedly increased the LC3-II protein expression in HFD-fed rats (*P*<0.01 or *P*<0.05). There was no difference between HFD-RES and HFD-CR groups in LC3-II protein expression of liver (*P*>0.05). Compared with the STD group, the SIRT1 protein expression of liver in the HFD group was slightly decreased (*P*>0.05), but the SIRT1 protein expression of liver in the HFD-RES group was obviously higher than the STD group and the HFD group (*P*<0.05 and *P*<0.01). Furthermore, there was also significant difference in the protein expression of SIRT1 between the HFD-CR group and the HFD group (*P*<0.05). Hepatic protein levels of GRP78 and CHOP were significantly increased in HFD group compared with STD group (*P*<0.01 and *P*<0.05). However, compared with HFD group, the hepatic protein levels of GRP78 and CHOP in HFD-RES group and HFD-CR group were significantly decreased (*P*<0.05 or *P*<0.01).

## Discussion

In this study, we compared the effects of resveratrol supplementation and caloric restriction on high-fat diet-induced non-alcohol fatty liver formation and hepatic steatosis by using male Wistar rats. The important finding of the present study was that resveratrol supplementation could mimic the beneficial effects of caloric restriction on lipid metabolism; both resveratrol supplementation and caloric restriction alleviated hepatic steatosis by up-regulating the SIRT1-autophagy pathway in high-fat diet-fed rats.

In animals, autophagy is a cellular process of lysosomal degradation that is vital for the healthy organism in that it removes excess or harmful proteins and organelles. Growing evidence from animal studies has reported that liver autophagy contributes to basic hepatic functions [35]. Another study reported that autophagy regulates the lipid content of the liver because the inhibition of autophagy increased TG and lipid droplets *in vitro* and *in vivo*; loss of autophagy decreased TG breakdown [13]. The above mentioned studies indicate that abnormal autophagy in the liver may contribute to lipid metabolism disorder of that organ. It is well established that nutritional deprivation through caloric restriction not only reduces the body weight of the individual but also activates SIRT1 and induces autophagy in cells. In addition, resveratrol is a classical dietary polyphenol and actives SIRT1[36]. Based on these reports, we used an HFD-induced rat model of hepatic steatosis to implement caloric restriction as a lifestyle modification and resveratrol as a drug therapy for lipid metabolism and study whether both forms of treatment act through SIRT1-mediated autophagy induction. Regarding the effects of caloric restriction on hepatosteatosis, a previous study reported that caloric restriction reversed hepatic steatosis and body weight with divergent hepatic metabolism in *db/db* mice [11], which was in agreement with our results. Moreover, long-term resveratrol treatment prevented dyslipidemia and hepatic steatosis in HFD-induced non-alcoholic steatohepatitis rodent models [28, 37]. For example, high dietary resveratrol intake (400 mg/kg bw) for 15 weeks significantly suppressed the body weight gain and fat accumulation in HFD-fed mice [19]. However, treated with resveratrol (400 mg/kg bw) for 8-week was observed to have a weak protective effect on the hepatic steatosis in HFD-fed Wistar rats [38]. Moreover, resveratrol (100 mg/rat/day) treatment for 8 weeks had no consistent therapeutic effect in alleviating manifest experimental steatohepatitis in HFD-fed rats [39]. The lack of consensus on the effect of resveratrol on HFD-induced lipid metabolism disorder and hepatic steatosis in rodent studies may have been due to different doses, varying diets and duration. In our study, we observed that an HFD resulted in lipid metabolism disorder in serum and lipid accumulation in the liver. We further found that long-term treatment with resveratrol (200 mg/kg bw) alleviated HFD-induced abnormal lipid metabolism and hepatosteatosis in rats. Compared with the resveratrol treatment, caloric restriction provided superior protection against HFD-induced hepatic steatosis and hepatocyte ballooning. The distinct effect of caloric restriction and resveratrol supplementation on HFD-induced formation has also been reported by Tauriainen E *et al*. [40]. Tauriainen E, found that both caloric restriction and resveratrol provided protection against diet-induced fatty liver formation, and caloric restriction showed better effects than resveratrol [40].

The present study further explored whether caloric restriction intervention and resveratrol supplementation alleviate hepatosteatosis by regulating autophagy in the liver. As LC3 and Beclin 1 have been established as useful signs for autophagy and p62/SQSTM1 is established as a selective substrate of autophagy as well as a marker of autophagic flux, we determined the hepatic mRNA and protein expression levels of *LC3* and *p62/SQSTM1* (autophagy markers) and the autophagy-related gene *Beclin 1* in liver. We found that an HFD decreased the *LC3* and *Beclin 1* levels in liver, whereas long-term with of resveratrol and calorie restriction deregulated the *LC3* level and reduced the level of *p62/SQSTM1*. These above data imply that the HFD-induced decline in autophagy substantially retards the clearance of excess free triglycerides, which in turn causes elevated levels of TG, TC and LDL, resulting in accumulation of fatty acids and lipid metabolic dysfunction aggregates and autophagy disorder in liver; resveratrol and caloric restriction treatment could reverse the decrease in autophagy and increase the autophagic flux of the liver in HFD-fed rats. SIRT1, an NAD^+^-dependent histone deacetylase, plays a vital role in metabolism and influences aspects of hepatic lipid metabolism. In addition, SIRT1 plays an essential role in protecting against HFD-induced metabolic damage [41, 42]. In animal studies, the sirtuin system can be influenced by caloric restriction and resveratrol [19, 43]; and a randomized trial study on a healthy human population demonstrated that caloric restriction and resveratrol supplementation significantly increased plasma concentrations of SIRT1[44]. To elucidate whether HFD would affect the SIRT1 level in the liver and whether the clearance of hepatic lipid accumulation treated by caloric restriction and resveratrol in HFD-fed rats required the SIRT1-mediated autophagy pathway, we further determined the expression of SIRT1 in the liver. Previous studies have reported that resveratrol increases the mRNA expression of hepatic SIRT1 in high-fat-fed mice [37]; and regulates human adipocyte number and function in a SIRT1-dependent manner [45]. In this study, we observed that HFD slightly reduced the mRNA and protein expression of SIRT1 in liver; however, resveratrol supplementation and caloric restriction increased the mRNA and protein expression of SIRT1 in liver.

Recently, ER stress has been thought to play a crucial role in lipotoxicity and inflammation, which are linked to both obesity and hepatic steatosis [46]. A previous study reported that resveratrol could protect against hepatic steatosis and ER stress in HFD fed rats [28]. Moreover, caloric restriction was shown to decrease ER stress in the liver and adipose tissue and improve hepatic insulin action in ob/ob mice [47]. To seek further evidence for the causal relationship of resveratrol and caloric restriction treatment to hepatic ER stress, we measured hepatic ER stress markers (GRP78 and CHOP) and the ER stress-associated protein PERK in four groups. In the present study, we found an increase in ER stress markers in the hepatocytes of HFD-fed rats, reflecting ER overload. In contrast, GRP78 and CHOP levels are commonly decreased in resveratrol-treated and caloric restriction-treated rats, showing the ER-restoring function of resveratrol and caloric restriction treatment. Interestingly, the impaired autophagic flux in the liver in both NAFLD patients and murine models of NAFLD can induce elevated ER stress and lead to apoptosis [48]. A link between impaired autophagic flux and increased endoplasmic reticulum stress was also observed in HFD-fed rats in this study. Together, therapies aimed at restoring autophagic flux and the subsequent ER stress might attenuate the progression of NAFLD.

In the present study, we observed that body weight, body weight gain, liver weight/body weight ratio and visceral fat coefficient were markedly increased in HFD-fed rats. It has been reported that resveratrol treatment (30 mg/kg/day) did not affect body weight but markedly decreased the liver weight of HFD-fed mice[36]; chronic caloric restriction (approximately 70% of the intake of the HFD-fed group) significantly reduced body weight and body weight gain in HFD-fed mice [18], which is in agreement with our study. In this study, after 18 weeks of caloric restriction and resveratrol treatment, the HFD-induced body weight gain of rats was also markedly decreased, and the body weight gain and visceral fat coefficient of the rats treated with caloric restriction were lower than those of the rats treated with resveratrol. These data suggested that caloric restriction for diet-induced obesity achieves more sustainable weight loss and visceral fat loss than is observed with pharmacological therapy. It has recently been reported that weight loss achieved by bariatric surgery (sleeve gastrectomy) ameliorates non-alcoholic fatty liver disease partly by increasing hepatic autophagy in high-fat diet-induced obese rats [49], which is in accordance with the results observed in the present study. This indicates that both caloric restriction and sleeve gastrectomy can increase hepatic autophagy and increase weight loss by reducing total energy intake. As for total energy intake, we reported here that chronic caloric restriction treatment could significantly reduce total energy intake, which is in accordance with the decreased body weight gain of HFD-fed rats. The above results indicate that reducing energy intake through dietary caloric restriction may be more effective than using resveratrol treatment to reduce weight.

In summary, this study reveals that long-term moderate caloric restriction (30%) intervention and resveratrol (200 mg/kg/day) supplementation improved lipid metabolism disorder and attenuated fat accumulation in the liver tissues of HFD-fed rats by up-regulating the SIRT1-autophagy signaling axis. Therefore, the present study suggests that humans could achieve beneficial metabolic effects through lifestyle modification (caloric restriction) and resveratrol supplementation, which would act by regulating the SIRT1-autophagy pathway and its downstream processes.

## Supporting information

S1 Table Body weight data for 18-week. (DOC)

S2 Table Body weight gain data for 18-week. (DOC)

S3 Table Liver weight and body weight ratio data. (DOC)

S4 Table Visceral fat coefficient data. (DOC)

S5 Table Total energy intake data. (DOC)

S6 Table Lipid in serum and liver data. (DOC)

## Abbreviations

CR: caloric restriction
RES: resveratrol
HFD: high-fat diet
ER stress: endoplasmic reticulum stress
LC3: microtubule-associated protein 1A/1B-light chain 3
SIRT1: sirtuins 1
PERK: protein kinase RNA-like endoplasmic reticulum kinase
GRP78: glucose-regulated protein 78/immunoglobulin-heavy-chain binding protein
CHOP: CCAAT/enhancer-binding protein- homologous protein
