# Supplementary material for: Resveratrol and caloric restriction prevent hepatic steatosis by regulating SIRT1-autophagy pathway and alleviating endoplasmic reticulum stress in high-fat diet-fed rats
Source: PLoS One. 2017 Aug 17;12(8):e0183541. doi: 10.1371/journal.pone.0183541 (PMC5560739; doi:10.1371/journal.pone.0183541)
Supplement: S1 Table — (DOC) [file pone.0183541.s001.doc]

**S1 Table. Body weight data for 18-week (Mean)**

| Week | STD group | HFD group | HFD-RES group | HFD-CR group |
| --- | --- | --- | --- | --- |
| 0 | 199.5 | 197.3 | 193.4 | 192.3 |
| 3 | 338.0 | 366.1 | 358.2 | 320.6 |
| 6 | 447.3 | 475.0 | 462.5 | 423.2 |
| 9 | 462.7 | 543.4 | 514.4 | 496.5 |
| 12 | 487.7 | 573.2 | 532.2 | 503.7 |
| 15 | 515.0 | 601.0 | 569.9 | 523.2 |
| 18 | 544.5 | 632.4 | 578.6 | 531.8 |
