# Supplementary material for: Resveratrol and caloric restriction prevent hepatic steatosis by regulating SIRT1-autophagy pathway and alleviating endoplasmic reticulum stress in high-fat diet-fed rats
Source: PLoS One. 2017 Aug 17;12(8):e0183541. doi: 10.1371/journal.pone.0183541 (PMC5560739; doi:10.1371/journal.pone.0183541)
Supplement: S2 Table — (DOC) [file pone.0183541.s002.doc]

**S2 Table. Body weight gain data for 18-week (Mean±SD)**

| STD group | HFD group | HFD-RES group | HFD-CR group |
| --- | --- | --- | --- |
| 327.3±40.98 | 387.5±20.97 | 348.3±31.25 | 334.3±28.24 |
