# Supplementary material for: Resveratrol and caloric restriction prevent hepatic steatosis by regulating SIRT1-autophagy pathway and alleviating endoplasmic reticulum stress in high-fat diet-fed rats
Source: PLoS One. 2017 Aug 17;12(8):e0183541. doi: 10.1371/journal.pone.0183541 (PMC5560739; doi:10.1371/journal.pone.0183541)
Supplement: S3 Table — (DOC) [file pone.0183541.s003.doc]

**S3 Table. Liver weight and body weight ratio data for 18-week (Mean±SD)**

| STD group | HFD group | HFD-RES group | HFD-CR group |
| --- | --- | --- | --- |
| 2.12±0.19 | 2.47±0.15 | 2.51±0.27 | 2.31±0.23 |
