# Supplementary material for: Resveratrol and caloric restriction prevent hepatic steatosis by regulating SIRT1-autophagy pathway and alleviating endoplasmic reticulum stress in high-fat diet-fed rats
Source: PLoS One. 2017 Aug 17;12(8):e0183541. doi: 10.1371/journal.pone.0183541 (PMC5560739; doi:10.1371/journal.pone.0183541)
Supplement: S4 Table — (DOC) [file pone.0183541.s004.doc]

**S4 Table. Visceral fat coefficient data for 18-week (Mean±SD)**

| STD group | HFD group | HFD-RES group | HFD-CR group |
| --- | --- | --- | --- |
| 2.38±0.25 | 5.32±0.54 | 4.87±0.29 | 3.95±0.38 |
