# Supplementary material for: Resveratrol and caloric restriction prevent hepatic steatosis by regulating SIRT1-autophagy pathway and alleviating endoplasmic reticulum stress in high-fat diet-fed rats
Source: PLoS One. 2017 Aug 17;12(8):e0183541. doi: 10.1371/journal.pone.0183541 (PMC5560739; doi:10.1371/journal.pone.0183541)
Supplement: S5 Table — (DOC) [file pone.0183541.s005.doc]

**S5 Table. Total energy intake data for 18-week (Mean, 10^3^ KJ)**

| STD group | HFD group | HFD-RES group | HFD-CR group |
| --- | --- | --- | --- |
| 35.51 | 47.82 | 49.19 | 36.51 |
| 37.90 | 47.75 | 47.83 | 36.83 |
| 37.41 | 46.52 | 47.28 | 37.13 |
| 39.59 | 50.82 | 44.78 | 37.08 |
| 35.77 | 48.05 | 47.43 | 36.52 |
