# Supplementary material for: Resveratrol and caloric restriction prevent hepatic steatosis by regulating SIRT1-autophagy pathway and alleviating endoplasmic reticulum stress in high-fat diet-fed rats
Source: PLoS One. 2017 Aug 17;12(8):e0183541. doi: 10.1371/journal.pone.0183541 (PMC5560739; doi:10.1371/journal.pone.0183541)
Supplement: S6 Table — (DOC) [file pone.0183541.s006.doc]

**S6 Table. Lipid in serum and liver data for 18-week (Mean±SD)**

| [Parameter](http://www.so.com/link?url=http%3A%2F%2Fdict.youdao.com%2Fsearch%3Fq%3Dparameter%26keyfrom%3Dhao360&q=参数+英语&ts=1491638878&t=a3950c0ed6a7ceab7768c38172a146d)s | STD group | HFD group | HFD-RES group | HFD-CR group |
| --- | --- | --- | --- | --- |
| Serum TG | 0.76±0.11 | 1.24±0.16 | 1.00±0.10 | 0.89±0.10 |
| Serum TC | 1.61±0.14 | 2.12±0.17 | 1.64±0.09 | 1.48±0.12 |
| Serum HDL | 1.40±0.17 | 0.71±0.10 | 1.18±0.15 | 1.30±0.13 |
| Serum LDL | 0.33±0.06 | 0.64±0.07 | 0.51±0.11 | 0.50±0.06 |
| Hepatic TG | 21.17±1.13 | 31.66±3.63 | 23.80±2.09 | 23.95±2.04 |
| Hepatic TC | 12.85±1.97 | 24.66±1.56 | 22.02±1.80 | 19.63±2.53 |
